# Identifying enriched drug fragments as possible candidates for metabolic engineering

**DOI:** 10.1186/s12920-016-0205-6

**Published:** 2016-08-10

**Authors:** Sunandini Sharma, Kritika Karri, Ishwor Thapa, Dhundy Bastola, Dario Ghersi

**Affiliations:** School of Interdisciplinary Informatics, University of Nebraska at Omaha, 1110 South 67th Street, Omaha, 68182 NE USA

**Keywords:** Metabolic engineering, Small molecules, Natural products, Chemoinformatics

## Abstract

**Background:**

Fragment-based approaches have now become an important component of the drug discovery process. At the same time, pharmaceutical chemists are more often turning to the natural world and its extremely large and diverse collection of natural compounds to discover new leads that can potentially be turned into drugs. In this study we introduce and discuss a computational pipeline to automatically extract statistically overrepresented chemical fragments in therapeutic classes, and search for similar fragments in a large database of natural products. By systematically identifying enriched fragments in therapeutic groups, we are able to extract and focus on few fragments that are likely to be active or structurally important.

**Results:**

We show that several therapeutic classes (including antibacterial, antineoplastic, and drugs active on the cardiovascular system, among others) have enriched fragments that are also found in many natural compounds. Further, our method is able to detect fragments shared by a drug and a natural product even when the global similarity between the two molecules is generally low.

**Conclusions:**

A further development of this computational pipeline is to help predict putative therapeutic activities of natural compounds, and to help identify novel leads for drug discovery.

## Background

A crucial factor for realizing the promises of precision medicine is the availability of novel and safe drugs to modulate the increasing number of targets that are being identified. Of all the medical branches, oncology is posed to be among those that could benefit the most from a new array of therapeutics [1].

Despite substantial progress in understanding the molecular basis of human cancers, there is still a pressing need for more effective, rational and personalized treatments. A few drugs for specific cancer types have achieved a good degree of selectivity with relatively low toxicity, but for the vast majority of human cancers, standard chemotherapy regimens (with their related toxicity) remain the only viable option. However, the situation is rapidly changing.

Breakthroughs in cancer genomics are now leading to the identification of new actionable targets [2], opening up unprecedented opportunities for personalized treatment. As a result of our improved understanding of cancer biology, with some notable exceptions the search for “silver bullet” therapies has now largely been replaced by a quest for novel targets that can be simultaneously modulated by combinatorial therapies [1, 3], akin to what has been accomplished for the treatment of HIV infections [4]. As a result, a vast number of suitable new drugs will soon be required to modulate a large array of cancer targets. Another area where the availability of new effective drugs is becoming a pressing need is the treatment of infectious diseases, as antibiotic-resistant bacteria are becoming more widespread and are a cause for serious concern [5].

The natural product derived structure plays a significant role in the discovery of novel pharmaceutical agents and/or bioactive molecules. The anti-diabetic activity in lupins has been attributed to quinozolidine alkaloids [6] and a review of the literature shows many such examples of natural products as sources of new drugs [7] including Paclitaxel, which is one of the most widely prescribed anticancer drugs on the market. Most of the natural products are biologically active and have favorable absorption, distribution, metabolism, excretion and toxicology properties. Plants are often the predominant source for the discovery of natural products due to the relative ease of access. However, more recently microbial as well as marine sources have been identified as alternative resources, particularly for antibiotics [8]. Several databases of natural products have been published and reviewed [9–12].

Although many pharmaceutical companies emphasize high throughput (HTP) screening of combinatorial libraries, natural products continue to provide enormous structural and chemical diversity to guide the careful design of drug-like leads. More importantly, the products of HTP screening often do not interact well with biomolecules and induce unexpected and possibly severe side effects. Therefore, over the years (since 1980) only 2 drugs obtained through the HTP screening have been approved by the FDA, while over 85 drugs are either natural products-based or compounds derived from them [13, 14]. In the past decade, several databases focusing on the collection of medically important natural products and medicinal herbs have been established [10, 11] and the use of computer aided drug design including virtual screening of large databases has become an important part of the drug discovery process [15].

Pharmaceutically relevant natural products are of low molecular weight and often restricted to special plant families. While these compounds are not important for the primary metabolism of the plants, they are found to be important for their survival in a given environment. Therefore, medicinally important plants are often collected from the wild or their natural habitat and are more likely to be endangered due to severe over collection [16]. Unfortunately, we still have limited knowledge about plant secondary metabolism, its regulation, molecular mechanisms concerning gene expression and rate-limiting enzymes found within a diverse network of biosynthetic pathways in living organisms.

Obtaining a drug completely from a plant source is often a difficult process, as the yield from the natural source may be small and the extraction process can be complex [17]. The solution to these problems is *partial chemical synthesis* or *semi synthesis*, where the aim is to extract only a biosynthetic intermediate or a bioactive fragment of the lead compound, which can then be developed into a drug using conventional synthesis [18]. This approach has several advantages: first, a biochemical intermediate can be more easily extracted with higher yield than the final product; second, it may be possible to synthesize analogues of the final product [19, 20].

The literature provides numerous examples of chemical fragments originating from natural sources which have been used for pharmaceutical purposes. For example, according to Lahlou et al. [21] the widely prescribed anticancer drug paclitaxel was manufactured by extracting 10-deacetylbaccatin from the needles of the yew tree, followed by a four-stage synthesis [21, 22]. Another example is provided by therapeutic drug fragments in plants with anti-fertility properties, which can be used as intermediates in the synthesis of contraceptive drugs from the natural source [23].

With the development of the fragment-based method described in this study, it is now possible to determine potentially important structures in natural products *in silico*, which may be investigated further to determine their pharmaceutical value as lead or intermediate compound, and potentially produced by cells cultivated in vitro utilizing plant biotechnology methods. To the best of our knowledge, this is the first time that a fragment-based approach using enrichment analysis is applied to identify potentially important chemical fragments in natural products.

## Methods

### Obtaining and representing drugs and natural products

The DrugBank database [24] (version 4.1) was used to obtain information on drugs that were approved for therapeutic use in at least one country. The initial set of drugs contained 1554 molecules. Natural products were obtained from the SuperNatural II database [12], containing 325,508 molecules.

Drugs and natural products were represented using the SMILES system [25], a widely used notation that makes it possible to encode chemicals as ASCII strings. SMILES strings for drugs and natural products were directly obtained from the DrugBank and SuperNatural II databases, respectively.

### Fragmenting the molecules

Both drugs and natural products were fragmented with the fragment program, part of the molBLOCKS suite [26], which breaks molecules along chemically important bonds and returns the corresponding fragments (or putative building blocks). The list of chemical bonds that were used by the program to fragment the molecules is shown in Fig. 2, and is based on Lewell et al. [27]. The minimum size for a fragment was set to four atoms, and the fragmentation was carried out with the “extensive” flag turned on, which yields all possible fragments that can be generated given the list of chemical bonds of interest [26].

It is noteworthy to mention that the fragmentation rules are encoded as SMARTS (SMiles ARbitrary Target Specification), an extension to the SMILES notation created by Daylight Chemical Information System, Inc. and widely used in computational chemistry. Using SMARTS patterns the particular bonds that are to be cleaved are encoded as regular expressions, making it straightforward to add other cleavable bonds to the fragmentation rules.

### Clustering fragments

Drug fragments obtained as described above were clustered with the analyze [26] program using standard parameters. In order to compute the fragment similarity for clustering, the program converts the fragment to a fingerprint representation, based on linear segments of up to 7 atoms in length (FP2 fingerprints [28]). The fingerprints are stored as bit arrays, where the presence or absence of a particular linear segment is represented by a 1 or 0, respectively. The FP2 fingerprint representation is obtained via the Open Babel library (http://openbabel.org/wiki/Tutorial:Fingerprints). Then, the Tanimoto coefficient *T*_*s*_ between two fragments *x* and *y* is computed as:

1 $$ T_{s} = \frac{{\sum\nolimits}_{i} X_{i} \land Y_{i}}{{\sum\nolimits}_{i} X_{i} \lor Y_{i}}   $$

where *X* and *Y* are the bit array representations of the linear segments found in fragment *x* and *y*, respectively, and ∧ and ∨ are the bitwise *and* and *or* operators.

The analyze program computes pairwise similarities between fragments and converts them to a graph representation, where an edge between fragments indicates a pairwise Tanimoto greater than the chosen threshold, which was set to 0.7 in this study. Subsequently, the program extracts the connected components of the graph, and selects the representative element for each cluster as the fragment with the highest average similarity against all the other fragments in the cluster.

### Extracting enriched fragments for each ATC code

In order to assign functional categories to drugs, we used the Anatomical Therapeutic Chemical (ATC) classification system (http://www.whocc.no/atc/structure_and_principles), a widely used nomenclature that organizes drugs according to the organ or system which they modulate and their therapeutic properties. The ATC code system is hierarchically organized into five levels of increasing specificity. We considered the second level, which describes the therapeutic main groups. We note that a single drug can be annotated with multiple ATC codes, if it has multiple therapeutic indications. For this study, to get meaningful statistics we selected all the ATC codes that annotated at least 10 distinct drugs.

Enrichment analysis was carried out in order to identify the specific fragments (or clusters of fragments) that appear in a set of molecules more frequently than expected by chance, given a background distribution. In this study the background was represented by the union of all approved drugs.

The analyze program uses the hypergeometric distribution to model the probability of obtaining a number of fragments (or clusters of fragments) equal to or greater than the observed by chance alone:

2 $$ P(X \ge k) = \sum\limits_{x=k}^{K}\frac{\binom{K}{x} \binom{N-K}{n-x}}{\binom{N}{n}}  $$

where *N* is the total number of fragments; *K* is number of fragments of the given type; *n* is the total number of fragments in the main set; and *x* is the total number of fragments of the given type in the main set.

The program returns both uncorrected *p*-values and False Discovery Rate (FDR) corrected *p*-values, obtained with the procedure of Benjamini-Hochberg [29]. In this study we selected fragments that were enriched with an FDR <0.05.

### Comparing enriched fragments in the drug dataset against fragments from natural compounds.

The final step of the pipeline involves the comparison between enriched fragments from the drug dataset against fragments obtained from the natural compounds set. In order to calculate the pairwise similarity between each of the enriched drug fragments and each of the fragments from natural compounds we used the Tanimoto coefficient (see Eq. 1). To carry out the calculations we wrote an in-house program that uses the Python API [30] of the OpenBabel library [28], and retained the drug fragment–natural product fragment pairs that had a Tanimoto similarity >0.9.

### Computational requirements for enrichment analysis and fragment comparison.

The most time-consuming step of the pipeline is represented by the pairwise fragment comparison, which took approximately 12 h on a 24-core machine. Fragmentation of the 325,509 molecules found in the SuperNatural II database took approximately eight hours on a 24-core machine, bringing the entire analysis to roughly 20 h.

### Biosynthesis pathway annotation.

We used the online SMILE converter program (https://cactus.nci.nih.gov/translate) to convert all chemical structures from SMILE format to MDL mol structural files, to be used later as an input for the pathway prediction algorithms. Chemspider (http://www.chemspider.com) [31], a free chemical structure database, was used to retrieve the IUPAC names and the chemical information for the enriched fragments.

The first step in pathway annotation was to determine the natural source and a possible biosynthesis pathway for the enriched fragments obtained from our pipeline. We used the Retropath webserver (http://www.jfaulon.com/category/retropath/) [22] and submitted each enriched fragment as an input query in MDL mol structural format. The output from the Retropath webserver consisted of a feasible biosynthetic pathway from the natural source, including the names of the enzymes catalyzing the reactions.

The next step in pathway annotation was to determine the synthesis pathway from fragments to drug compound. To accomplish this task, we used the Pathpred webserver (http://www.genome.jp/tools/pathpred), which predicts the synthesis pathway given the substrates and the final product. The Pathpred webserver is linked to the KEGG database and the user can input a query compound in the MDL mol file format, in the SMILES representation, or using the KEGG compound/drug identifier. The enriched fragments with known biosynthesis pathway (obtained from Retropath) were given to Pathpred as initial substrate. The drug compounds associated with the enriched fragment obtained from the enrichment pipeline were given as the final product in order to get possible synthetic routes between the fragment and drug.

## Results

### A computational pipeline to systematically compare functionally relevant drug fragments and natural products

We set out to systematically compare approved drugs obtained from the DrugBank database [24] against a large collection of natural products, assembled in the SuperNatural II database [12]. The novelty of our approach consists first in extracting the fragments that are statistically overrepresented in each pharmacological category, and then in comparing those fragments against the ones derived from the natural compounds.

**Fig. 1:**
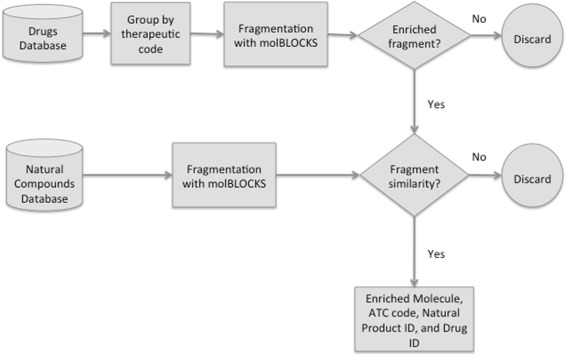
Simplified overview of the pipeline. Each approved drug (obtained from Drugbank [24]) is assigned a therapeutic class using the ATC nomenclature. The drugs are then broken down into fragments using the molBLOCKS software [26], and enrichment analysis is performed on each therapeutic class to identify statistically overrepresented fragments (FDR <0.05). Each overrepresented fragment is then compared against similarly obtained fragments from a database of natural compounds [12] (see Materials and Methods for further details)

The rationale behind this approach is twofold. On the one hand, chemical fragments capture important properties of the full molecules, and on the other hand they may be shared by otherwise globally dissimilar molecules, which might go undetected when using a global similarity measure. The pipeline is briefly outlined in Fig. 1, which shows the main steps of the procedure. More details are found in the Materials and Methods section of the paper.

**Fig. 2:**
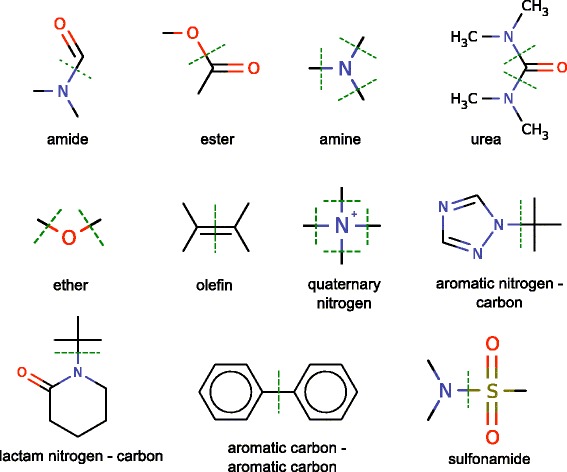
RECAP rules used to fragment drugs and natural products. The 11 eleven types of chemical bonds depicted above (*green dashed lines*) indicate the potential sites that can be broken in the small molecules, resulting in smaller fragments. These 11 fragmentation rules were derived from Lewell et al. [27], and capture chemically relevant synthetic reactions that combine building blocks into more complex molecules

In order to fragment the molecules we used the molBLOCKS suite [26] with the RECAP rules [27] (Fig. 2), which allow us to break small molecules apart along chemically important bonds. It is noteworthy to mention that in several cases no fragmentation rule applies to a small molecule, which is then left as it is and treated as a whole fragment. In our initial dataset of 1,543 approved drugs we were able to fragment 949 (62 %) of the drugs. The remaining ones, for which no fragmentation RECAP rule applies, were treated as one fragment. In the case of natural products, the fragmentation rules applied to 174,156 (54 %), and the remaining molecules were treated as one fragment.

Subsequently, we grouped drugs by Anatomic Therapeutic Code, which gives the therapeutic group of a drug (e.g., “L01” stands for “Antineoplastic Agents”, “C03” for “Diuretics”, etc.). Multiple membership of a drug in several ATC groups was allowed if the drug was annotated in DrugBank with multiple ATC codes. We ended up with 40 ATC groups, each containing at least 10 distinct drugs. For each ATC group, we performed clustering of the fragment followed by enrichment analysis with the molBLOCKS suite, extracting statistically overrepresented fragments for each group, with an FDR <0.05. The total number of enriched fragments across all therapeutic groups was 141.

In the last step of the pipeline, we systematically compared the enriched fragments from each ATC group against the fragments obtained from the natural compounds, and retained for further analysis all the pairs that had a Tanimoto similarity >0.9.

### Drugs and natural compounds are related at the fragment level in specific therapeutic groups

**Fig. 3:**
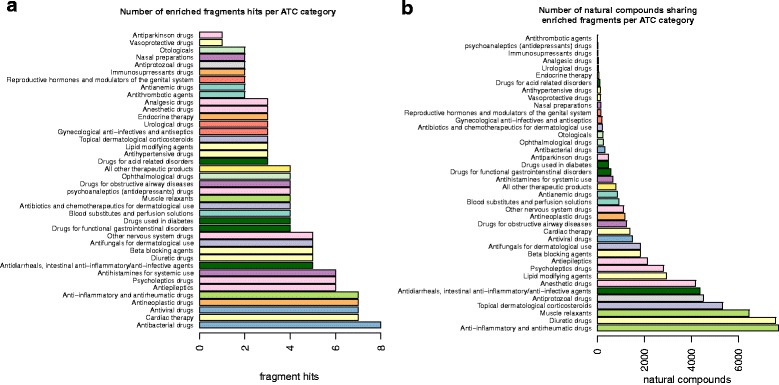
Distribution of enriched fragments and matching natural compounds per ATC code. Panel (**a**) shows the number of drug fragments that are enriched in given therapeutic categories (ATC codes) that have at least one matching fragment in the set of natural compounds. Panel (**b**) shows the total number of natural compounds whose fragments match one or more of the enriched drug fragments in each therapeutic category

We considered the number of fragments for each therapeutic group with at least one matching fragment in the natural products dataset, obtaining the distribution shown in Fig. 3. The top-ranking group was represented by the antibacterial drugs, followed by drugs active on the cardiovascular system, antiviral drugs, antineoplastic drugs, and anti-inflammatory drugs. The prominence of antibacterial drugs in this list is consistent with the importance that natural products have had in the development of antibiotics [32].

An alternative way of analyzing these data is to consider the number of natural products whose fragments match at least one of the fragments in each therapeutic group. The results are shown in Fig. 3. The therapeutic group with the largest number of natural products is now the anti-inflammatory class, closely followed by diuretic drugs, muscle relaxants, and corticosteroids.

### Case studies

**Fig. 4:**
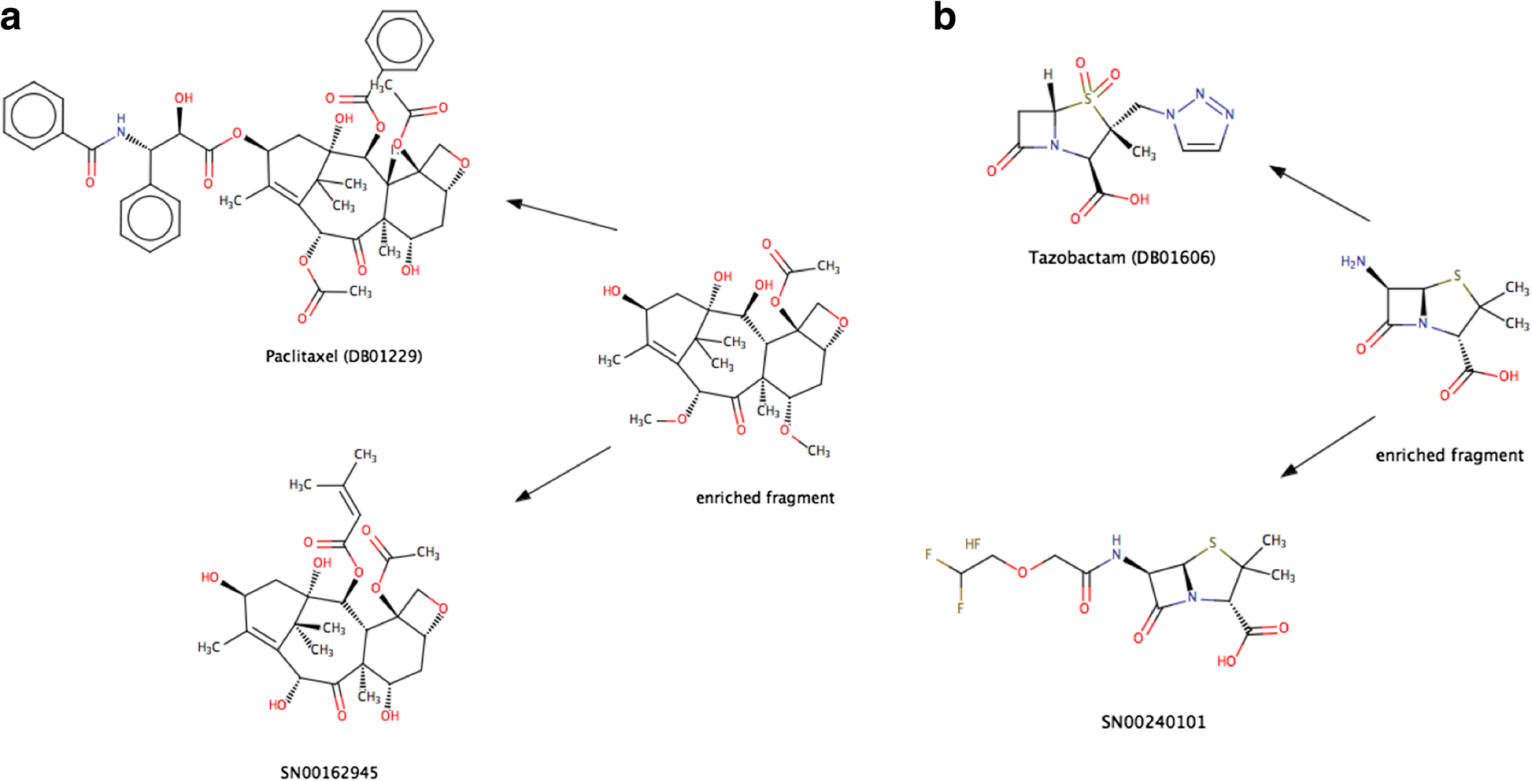
Examples of fragments shared by natural compounds and drugs in the absence of high global similarity. The two examples shown here illustrate how a fragment-based approach can automatically detect commonalities between molecules that are globally different. Panel (**a**) shows a tetracyclic fragment present both in a natural compound and in an anti-cancer agent (Paclitaxel). In spite of the common core shared by the two molecules, the Tanimoto similarity between the drug and the natural compound is relatively low (0.56). In panel (**b**), the beta-lactam ring is detected (which a small variation) in both an approved antibiotic (tazobactam) and a natural compound (SN0240101). However, the Tanimoto similarity between the natural compound and tazobactam is low (0.49)

In Fig. 4 we show two examples of fragments shared by a drug and a natural product in the context of low global similarity. One of the advantages of our fragment-based approach is the automatic identification of common and chemically important building blocks among molecules that may be globally dissimilar.

A proof of concept is given by the anticancer drug Paclitaxel and the natural product SN00162945 (Fig. 4), which share a tetracyclic core but have different substituents. In fact, Paclitaxel itself was first isolated from the bark of a yew, and belongs to the taxane family, whose members all share the core fragment shown in the figure (or a closely related variation). However, because of the different substituents in the two molecules, the Tanimoto coefficient between Paclitaxel and SN00162945 turns out to be only 0.56.

Another example that showcases the power of using fragments is shown in Fig. 4. The antimicrobial Tazobactam contains a *β*-lactam ring, which is the building block of a highly important group of widely prescribed antibiotics, including penicillin, cephalosporins and carbapenems, and it occurs in several natural compounds. As in the example of Fig. 4, Tazobactam has a low Tanimoto similarity (0.49) for the natural product SN00240101, in spite of the fact that they both share the *β*-lactam ring.

### Pairwise global similarity of drugs and natural products that share an enriched fragment

**Fig. 5:**
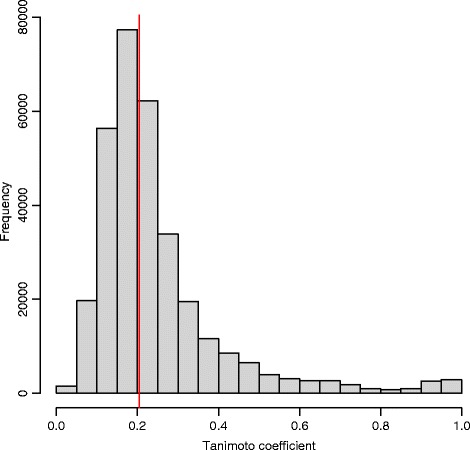
Distribution of pairwise Tanimoto similarity between drugs and natural products that share an enriched fragment. The distribution was obtained from 320,134 pairs of drugs/natural products. The *red line* indicates where the median of the distribution falls

The case studies discussed above suggest that the proposed fragment-based approach can capture local similarities between molecules that are otherwise globally different. In order to test this hypothesis systematically, we set out to compare the global similarity between drugs and natural products sharing an enriched fragment. We systematically computed the pairwise Tanimoto similarity between drugs and natural products that had at least one enriched fragment in common (defined as a Tanimoto similarity >0.90 between the enriched fragment and the natural product fragment), obtaining the distribution shown in Fig. 5 and containing 320,134 comparisons. The median of the distribution (indicated by a red line in Fig. 5) is 0.204, confirming that the shared enriched fragments often occur in globally different molecules.

### Biosynthetic pathway analysis

The computational fragmentation process yields fragments that are chemically plausible, but for which there is no guarantee to their existence in biological pathways as standalone molecules. To address whether some of the fragments could in fact be identified in biosynthetic pathways, we processed all 112 enriched fragments coming from approved drugs using the Retropath webserver. Retropath returned a total of nine fragments with a known biosynthetic pathway in a plant or microbial organism. The enriched fragments with a known biosynthetic pathway are shown in Table 1, which also provides the E.C. number, the IUPAC name of the enzyme that catalyzes the biosynthetic reaction leading to the fragment, and the organism source. Seven out of the nine fragments are found in plant sources, and the remaining two come from the fungi kingdom (*Rhodotorula glutinis* and *Acremonium chrysogenum*).

**Table 1:** Enriched fragments with SMILES code, IUPAC name, chemical structure, and source (organism)

Enriched fragment	IUPAC name	Structure	Source
CCCc1ccc(cc1)O	4-Propylphenol		*Brassica napus* (Rapeseed)
CCCCCCCCCCCC	Dodecane		*Pisum sativum* (pea)
CCCCCCCCC(=O)O	Nonanoic acid		*Pisum sativum* (pea)
C(=O)CCCC[N+]	5-Amino pentanal		*Arabidopsis thaliana* (thale cress)
O=c1[nH]c(=O)c2c(n1C)nc[nH]2	Caffeine		*Camellia irrawadiensis*
OC(=O)[C@H](Cc1c[nH]c2c1cccc2)N	L-Tryptophan		*Sinapis alba* (white mustard)
C/C=C/c1ccccc1	Styrene		*Saccharomyces Cerevisiae* (budding yeast)
Cc1c[nH]c(=O)[nH]c1=O	Thymine		*Rhodotorula glutinis*
O=C1[C@@H](N)[C@@H]2N1C(=C(CS2)C)C(=O)O	3-Methyl-7-aminoceph-3-em-4-carboxylic acid		*Acremonium chrysogenum*

### Case studies

We next addressed whether it is possible to identify biosynthetic pathways in plants and microorganisms that can potentially turn an enriched fragment into a known drug. As discussed in the Methods section, we used the Pathpred webserver to extract known biosynthetic pathways from an enriched fragment to a drug product. For five fragments (caffeine, 5-aminopentanal, glycine, styrene, and thymine) we could identify a biosynthetic pathway leading to the fragment and also biosynthetic pathways leading to drugs. Two case studies are discussed below.

**Fig. 6:**
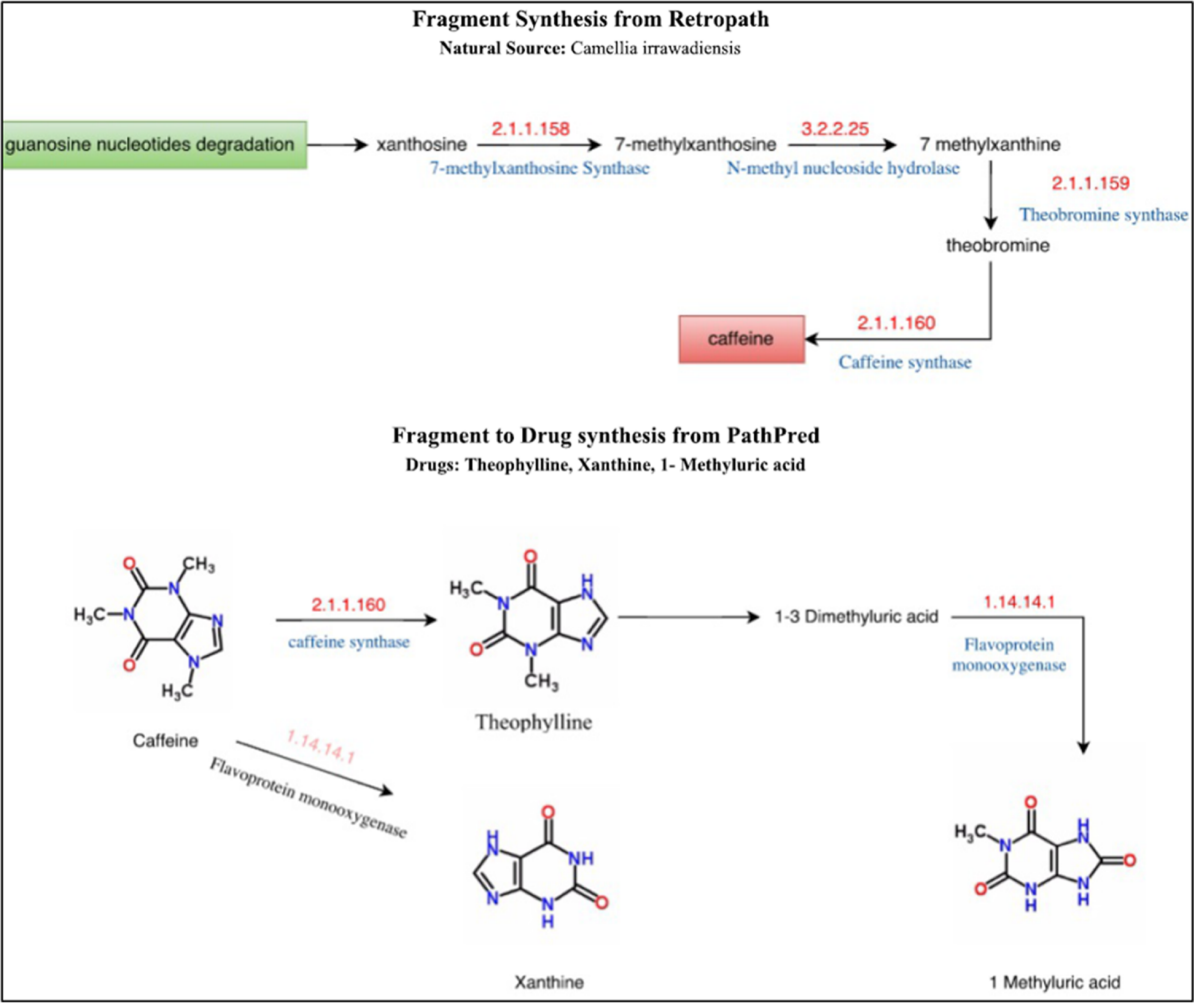
Biosynthetic pathway involving caffeine. Information about the biosynthetic pathway of the enriched fragment caffeine in C. irrawadiensis was obtained from Retropath (*top*). The biosynthetic pathways from caffeine to xanthine, theophylline, and 1-methyluric acid were obtained from Pathpred (*bottom*)

**Caffeine biosynthesis.** One of the enriched fragments for which Retropath could return a biosynthetic pathway was 1,3,7-trimethylxanthine, commonly known as caffeine, which is found in coffee plants (*Coffea arabica*) and young leaves of tea plant (*Camellia sinensis*, Table 1). Caffeine synthesis begins in these plants with xanthosine as the initial substrate, which is then converted into 7-methylxanthosine followed by a second methylation step which leads to the formation of theobromine. The final product is synthesized by the enzymes theobromine synthase (EC 2.1.1.159) and caffeine synthase (EC 2.1.1.160), which convert 7-methylxanthine to theobromine and theobromine to caffeine, respectively (Fig. 6). Our fragment enrichment pipeline identifies caffeine as significantly enriched in drugs active on the respiratory system, including theophylline (DrugBank ID: DB00277), a drug which is used in the acute treatment of asthma. Pathpred provided the synthesis route between caffeine fragment and theophylline. Additionally, it also provided the synthesis pathway for other derivatives (1-methyluric acid and xanthine).

**Fig. 7:**
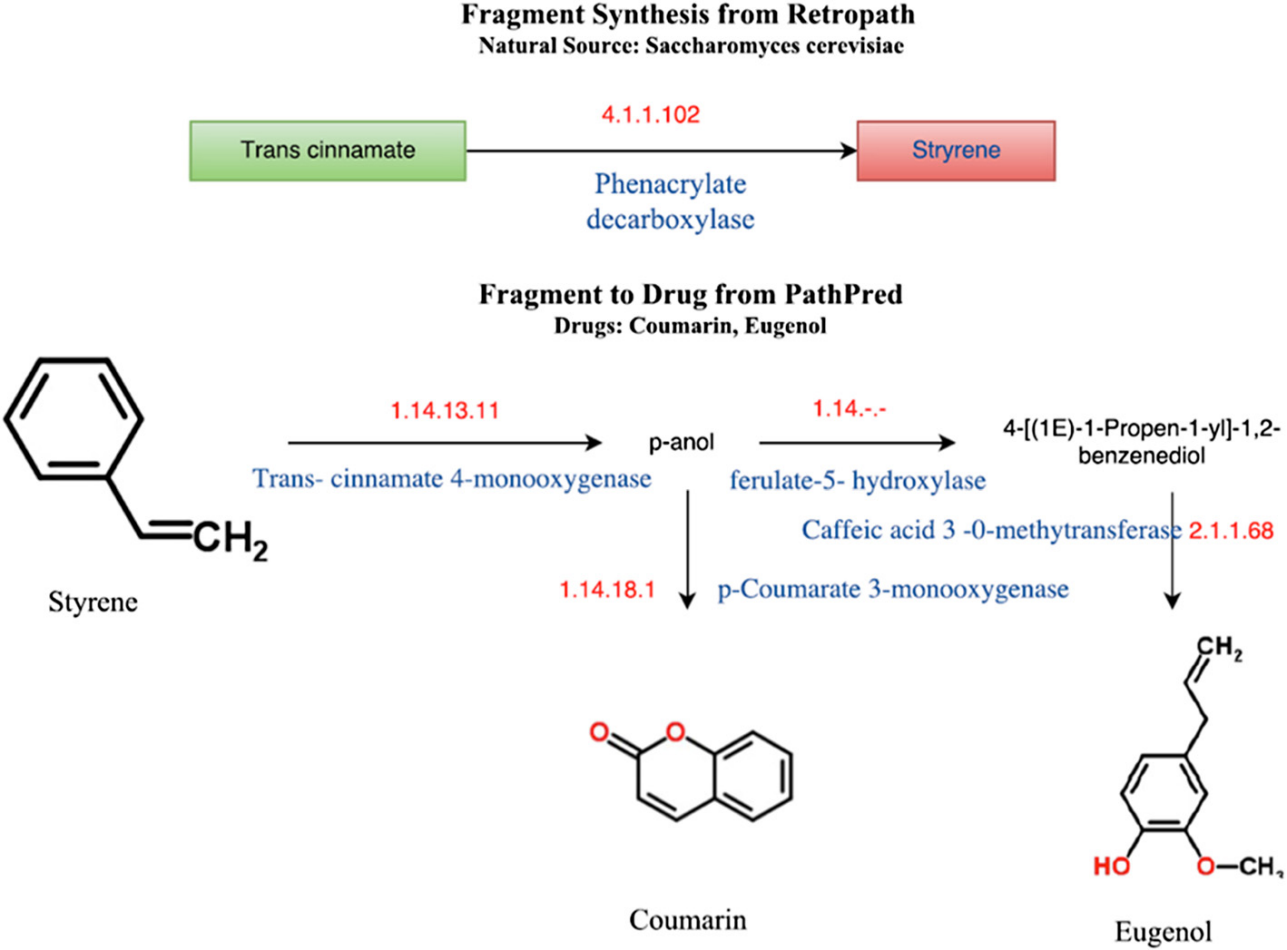
Biosynthetic pathways involving styrene. Information about the biosynthetic pathway of the enriched fragment styrene in *S. cerevisiae* irrawadiensis was obtained from Retropath (*top*). The biosynthetic pathways from styrene to coumarin and eugenol were obtained from Pathpred (*bottom*)

**Styrene biosynthesis.** For some enriched fragments we found a biosynthetic pathway in microorganisms. For example, the styrene fragment was found to have a biosynthesis pathway in *S. cerevisiae* (budding yeast). The enzyme ferulic acid decarboxylase (EC 4.1.1.M2) catalyzes the production of styrene from the substrate trans-cinnamate (Fig. 7). Using styrene as substrate in *Pathpred* we identified biosynthetic pathways for two drugs: Eugenol (DB09086) and Coumarin (DB00682). The enzyme trans-cinnamate 4-monooxygenase (EC 1.14.13.11) converts styrene to p-anol. Two different enzymes acts on p-anol: p-Coumarate 3-monooxygenase (EC: 1.14.18.1), which catalyzes the production of the Coumarin drug, and ferulate 5-hyroxylase, which converts converts p-anol to 4-[(1E)-Propen-1-yl]-1, 2- benzenediol. Finally, caffeic acid 3-O-methyltransferase (EC: 2.1.1.68) acts on 4-[(1E)-Propen-1-yl]-1,2- benzenediol to give Eugenol (Fig. 7). Eugenol has analgesic, local anesthetic, anti-inflammatory and antibacterial effects, and is widely used in dental care practice [33]. It also prevents oxidative changes in membrane and acts as an antioxidant [34]. The other product (coumarin) is used as an anti-coagulant. It also has anti-fungicidal and anti-tumor activities.

## Discussion and conclusion

The natural world as a source of highly diverse and complex chemicals has always been of value to synthetic chemists, and is becoming even more relevant today, given the output slump of the pharmaceutical industry. The pipeline introduced here allows to automatically detect relationships between small molecules using a fragment-based approach. Using a fragment-based approach is motivated by the fact that natural products are often assembled from independent building blocks via a chain of enzymatic reactions. These processes are somewhat similar to what is common practice in synthetic chemistry.

By first extracting statistically overly represented fragments for each therapeutic class we reduced the complexity of the approved drugs to a handful of chemical fragments that are likely to be responsible (at least in part) for the pharmaceutical activity of the given drugs, or are important as chemical scaffolds. Comparing these fragments against the fragments obtained from a large library of natural products allowed us to establish potential relationships between drugs and natural products even in the absence of high global similarity between the molecules. As an analogy, we could compare this fragment-based approach to a local sequence alignment procedure, which can identify highly similar protein domains among globally different protein sequences.

As a note of caution, we should mention that the choice of the Tanimoto similarity thresholds or the stringency of the fragment clustering step would affect the final results, in that more or less matching fragments would be found depending on how stringent the parameters that control the similarity are set to be. Unfortunately, there are no hard and fast rules to guide the user in the choice of parameters. However, as it is often the case in bioinformatics applications, the results should be interpreted as a guide to help design further experiments or perform more thorough literature searches. In this context, our pipeline could be used to ask the question of whether a natural product that happens to share a fragment with an antihypertensive drug does in fact have pressure lowering activity. Alternatively, the pipeline could be used to investigate whether a natural product shows potential as a lead compound for a given therapeutic indication.

In the future we plan to extend our pipeline by mining databases to automatically collect biosynthetic pathway information, and do more extensive analyses on the sources of natural compounds. Although this may not be possible for all compounds, databases like the “Universal Natural Product Database” [11] (contained in SuperNatural II) do include source information for several compounds. Combined with metabolic information on plant and microbial pathways, this will yield a better understanding of natural product synthesis. As shown by a pioneering study by Runguphan et al. [35], this could eventually lead to co-opting natural systems for engineering better drugs.
